# Treatment of Distal Third Humeral Shaft Fracture with Intramedullary Nail Combined with Anterior Minimally Invasive Plate Osteosynthesis

**DOI:** 10.1111/os.13893

**Published:** 2023-10-10

**Authors:** Gang Fu, Yichong Zhang, Shuyujiong Ke, Dengke Zhu, Jingxiang Wu, Dengbang Su, Hui Ge, Jianlong Chen, Yan Zhang MB, Fengfei Lin, Jianhai Chen, Renbin Li

**Affiliations:** ^1^ Department of Orthopaedics, Fuzhou Second Hospital, Fujian Trauma Orthopaedics Emergency and Rehabilitation Clinical Medical Research Center Fuzhou Trauma Medical Center Fuzhou China; ^2^ Department of Trauma & Orthopedics Peking University People's Hospital Beijing China

**Keywords:** Elbow, Fracture fixation, Humeral, Intramedullary nail, Minimally invasive, Plate

## Abstract

**Objective:**

The treatment of distal third humeral shaft fracture is difficult. Studies have shown that anterior minimally invasive plate has lower probability of complication and higher healing rate. However there is no applicable anatomical plate at present. This study is to investigate the clinical effect of intramedullary nail combined with anterior minimally invasive plate in the treatment of distal humeral shaft fractures.

**Methods:**

The data of 83 patients with lower humerus shaft fracture treated from September 2015 to January 2020 were analyzed. According to different treatment methods, they were divided into two groups: 40 patients were treated with intramedullary nailing combined with minimally invasive anterior plate fixation (group A), and 43 patients were treated with double plate fixation through posterior approach (group B). General preoperative data, operative time, intraoperative blood loss, total incision length, fracture healing time, shoulder and elbow visual analogue scale (VAS) score, Constant–Murley shoulder function score, Mayo elbow function score, and complications were recorded and compared between the two groups. Two independent sample *t*‐tests was used for follow‐up, age, BMI, operation time, intraoperative bleeding, total incision length, fracture healing time, Constant–Murley score and Mayo score, and rank sum test was used for VAS score of shoulder and elbow.

**Results:**

There was no significant difference in preoperative general data between the two groups (*p* > 0.05), indicating comparability. There were no significant differences in operation time, total incision length, fracture healing time, Constant–Murley shoulder function score at the last follow‐up, Mayo elbow function score, and shoulder and elbow VAS pain score between 2 groups (*p* > 0.05). The amount of intraoperative blood loss in observation group was 76.98 ± 16.46, which was significantly less than that in control group, and the difference was statistically significant (*p* < 0.01). There were no radial nerve injury, musculocutaneous nerve injury, incision infection and fracture nonunion in the observation group. In the control group, four cases of iatrogenic radial nerve injury, three cases of incision infection and three cases of fracture nonunion were found. The complication rate was 23.3% (10/43). There was statistical difference in the incidence of complications between the two groups (*p* < 0.01).

**Conclusion:**

A humeral intramedullary nail combined with an anterior minimally invasive plate in the treatment of distal humeral shaft fracture has the advantages of less soft tissue damage, less blood transfusion, high fracture healing rate and low risk of iatrogenic radial nerve injury, which is an effective method for clinical treatment of this type of fracture.

## Graphical abstract

We introduced a new method using a humeral intramedullary nail combined with an anterior minimally invasive plate in treating distal humeral shaft fracture, which has the advantages of less soft tissue damage, less blood transfusion, high fracture healing rate and low risk of iatrogenic radial nerve injury.

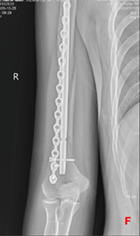

## Introduction

Lower humeral shaft fracture, also known as distal third humeral fracture, accounts for about 7% of humeral shaft fractures, 16% of humeral fractures and 3% of systemic fractures.^1^, ^2^ Due to the irregular anatomical morphology of the lower humerus, there is no standard surgical treatment so far.

An intramedullary nail is rarely used since the distal locking distance is limited.^3^, ^4^ A retrograde intramedullary nail of the humerus can be used for the treatment of lower humeral shaft fracture, but has the risk of iatrogenic supracondylar fracture and axillary nerve injury.^5^, ^6^ Plate fixation is the widely used method in treating humeral fractures. Due to the irregular shape and limited length of the distal end, the strength of a single plate through the anterior or lateral approach is unstable, increasing the risk of internal fixation failure.^7^ Double plate fixation has reliable strength and can realize early functional exercise, usually done through the posterior approach.^8^ However, the proximal end of the radial nerve needs to be exposed and separated, repeated traction during the operation increases the risk of iatrogenic radial nerve injury, reported as 16%–31.1%.^9^, ^10^, ^11^ At the same time, the nonunion rate is up to 2.8%–21%, due to the soft tissue stripping and blood supply damage.^12^ Some surgeons try to fix this with double plates through a medial‐anterior approach. Since the medial surgical approach needs to expose the ulnar nerve and brachial artery, the anatomical structure is relatively complex, so it is rarely used at present.

Studies have shown that the anterior minimally invasive plate has lower probability of iatrogenic radial nerve injury, less blood supply damage and higher healing rate.^13^ However, due to the limited length of distal end and irregular shape, there is no applicable anatomical plate at present. If a reconstruction plate is selected, only two or three effective screws can be inserted into the distal end of some fracture types, and the fixation strength is insufficient. Early functional exercise has the risk of failure.^7^ In this study we used humeral intramedullary nail combined with anterior minimally invasive plate fixation to treat lower humeral shaft fracture. This combination can not only increase the strength of fixation, but also reduce the risk of iatrogenic radial nerve injury. The purposes of this study were to analyze the data of patients with lower humeral shaft fractures and compare the clinical results between two different methods in treating lower humeral shaft fractures.

## Methods

### Inclusion and Exclusion Criteria

Inclusion criteria: (i) aged 20–60 years; and (ii) patients with distal third humeral shaft fracture. Exclusion criteria: (i) open fracture; (ii) radial nerve injury before operation; (iii) multiple fractures of ipsilateral limbs; (iv) patients with craniocerebral trauma who cannot cooperate with functional exercise after operation; (v) the distance from fracture line to joint surface less than 2cm; (vi) fractures that extend into the distal humeral metaphysis or articular surface; and (vii) osteoporosis.

### General Information

**FIGURE 1:**
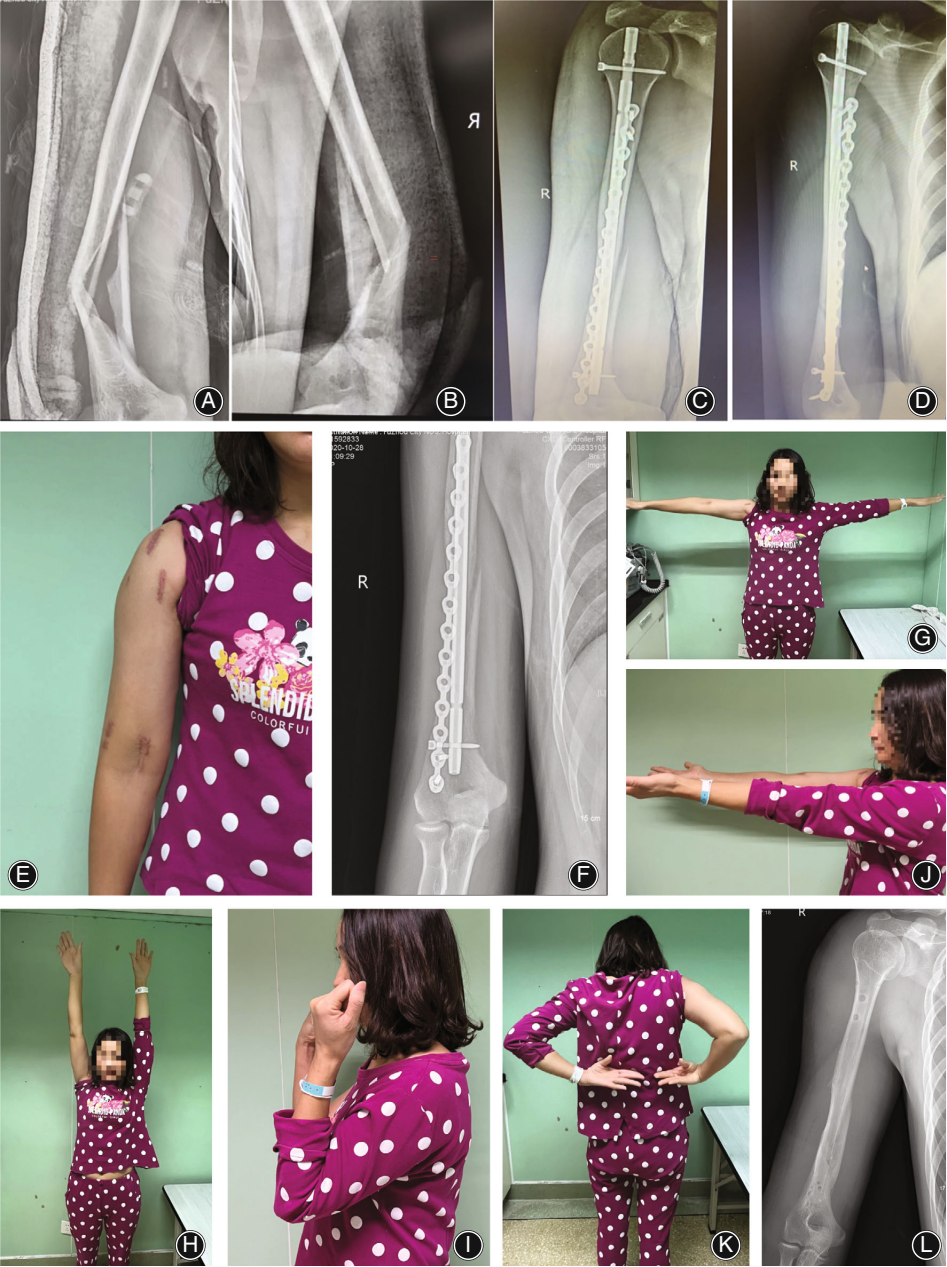
The patient was a 35 year old female with right elbow injury caused by falling. The anteroposterior and lateral films of right humerus before surgery (A, B): the lower humeral shaft was fractured with a wedge‐shaped segment on the inner side. AO classification: 12B2. On the third day after injury, surgery with a humeral intramedullary nail combined with an anterior minimally invasive plate was performed. The AP and lateral films of humerus were rechecked after surgery (C, D). During operation, alignment was restored after the insertion of a humeral nail. The medial bone mass of distal humerus was automatically reduced through the soft tissue hinge, and the soft tissue was protected. Surgical incision is shown in (E). The fracture healed 8 weeks after surgery (F). Function at 12 weeks after surgery (G–K). The internal fixation was removed 12 months after surgery (L).

Eighty three patients were included in this study from September 2015 to January 2020. All the patients signed informed consent and agreed to join this trail. They were then randomized into two groups before surgery double blindly using draw lots, patients with odd number (1, 3, 5, 7, 9) were included into Group A (intramedullary nail combined with anterior minimally invasive plate group) and even numbers (2, 4, 6, 8, 0) were included into Group B (posterior approach double plate group). In group A, there were 40 patients including 22 males and 18 females, the average age was 40.5 ± 9.9 years, fracture classification: 12 cases of type A, 25 cases of type B and 3 cases of type C. There were 43 cases in group B, including 24 males and 19 females; The average age was 38.9 ± 13.3 years. According to AO classification, there were 15 cases of type A, 26 cases of type B and two cases of type C. The general preoperative data of the two groups are shown in Table 1. This study was approved by the ethics committee of Fuzhou second hospital affiliated to Xiamen University (2020133), and the study was registered on Clinicaltrials as NCT03358173. All patients had been informed and signed the informed consent form (Figure 1).

**TABLE 1:** Comparison of gender, age, fracture AO classification, injury to operation time and follow‐up time between the study group and the control group.

Group	N	Gender (n)		Age	AO Classification			Days between injury and surgery	Follow‐up time (Month)
		Male	Female	(Years, *x* ± s)	A	B	C		
Group A	40	22	18	40.5 ± 9.9	12	25	3	3.04 ± 1.97	14.49 ± 3.36
Group B	43	24	19	38.9 ± 13.3	15	26	2	3.44 ± 0.50	13.83 ± 6.11
Statistic and *p* value	‐	0.963		−1.987	2.443			0.580	0.394
		0.521		0.991	0.891			0.519	0.674

## Surgical Method

### Humeral Intramedullary Nail Combined with Anterior Minimally Invasive Plate Fixation

#### Anesthesia and Position

The patient was in supine position and the injured shoulder was padded up. General anesthesia combined with brachial plexus block was used.

#### Approach

The proximal incision extended from the anterior edge of acromion to distal, entered between the anterior and middle bundle of deltoid, cut off the deltoid bursa and expose the supraspinatus tendon. The insertion point was located at the posterolateral side of bicep groove and about 1 cm inside the apex of greater tuberosity. It was cut sharply along the supraspinatus fiber to expose the bone of the humeral head, carefully protecting the supraspinatus tendon, then the guidewire was inserted then take intraoperative X‐ray with AP and outlet view to confirm the location.

#### Reduction and Fixation

The assistant maintained the elbow flexion for traction and reduction, and inserted the guidewire into distal cavity. After passing through, the reaming diameter was 1–1.5 mm larger than the nail, and the length was measured. The nail was locked at the distal end, the rotation alignment and knock back were adjusted according to the cortical thickness of fracture under fluoroscopy, then the proximal screws were locked. The distal incision extended about 3 cm from the center of cubital fossa to the proximal end, the biceps brachii were pulled inward exposing the brachial muscle, and attention was paid to protecting the musculocutaneous nerve on the surface of brachial muscle. The brachial muscle was split longitudinally on the medial and lateral sides of the brachial muscle to expose the bone cortex of the distal humerus. The reconstruction plate was selected and the proximal end was located above the spine of the humeral tubercle and the distal end was located above the coronal fossa. Two bicortical locking screws were inserted into the distal and proximal ends respectively. Fluoroscopy confirmed the position of the plate and the length of screws. After washing the wound, rotator cuff tissue was repaired and the wound was closed.

### Open Reduction and Double Plate Fixation via Posterior Approach

The patient was placed in a lateral position, and general anesthesia combined with brachial plexus anesthesia was used. The distal part of the nail used in this study (Hangwei, Shandong, China) were straightened with two locking holes, which can reach at least 2 cm above the olecranon fossa. The posterior approach of humerus was taken, entering from the muscle interval, separating and protecting the radial nerve carefully, marked with rubber belt, which is an important and difficult part of this procedure. The triceps brachii was pulled to expose the fracture end, the fracture was reduced and temporarily fixed with Kirschner wire under direct vision. A suitable plate was chosen and placed on the humeral shaft, locking screws were placed at both ends of the plate. Then a longer locking plate was placed on the back of humerus to form an angle of 90°. The position and length of plate were determined by C‐arm, then the incision was closed.

### Postoperative Treatment and Follow‐up

The rehabilitation exercise started 3 days after operation, such as repeated fist clenching, passive functional exercise and ice compression. Before discharge, patients were taught shoulder pendulum movement, wall climbing training, upper limb abduction, lifting and other functional exercises. Four weeks after operation, the passive exercise was mainly used, changing to partial active exercise at 4–8 weeks, and anti‐resistance training at 8–12 weeks. After discharge, outpatient follow‐up to guide patients was encouraged to further functional exercise.

### Observation Index

The operation time, intraoperative bleeding, total length of incision and fracture healing time were observed and recorded. Patients returned to the hospital for follow‐up at 1, 3, and 6 months after operation, and completed the Constant–Murley shoulder function score, Mayo elbow function score and visual analogue scale (VAS) pain score of shoulder and elbow at the last follow‐up. The postoperative complications were recorded, including radial nerve injury, musculocutaneous nerve injury, incision infection and fracture nonunion (a fracture that persists for a minimum of 9 months without signs of healing for 3 months).

### Statistical Method

We used SPSS statistical software (Version 22.0, IBM, Armonk, NY, USA). The follow‐up time, age, body mass index (BMI), operation time, intraoperative bleeding, total incision length, fracture healing time, Constant–Murley score and Mayo score all had normal distribution with homogeneous variance, with *X ± s* indicates. Shoulder VAS score and elbow VAS score had non‐normal distribution, expressed as median (quartile) [M (P25, p75)]. Two independent sample *t*‐tests were used for the comparison between the two groups of normal data (follow‐up time, age, BMI, operation time, intraoperative bleeding, total incision length, fracture healing time, Constant–Murley score and Mayo score), and rank sum test was used for non‐normal data (shoulder and elbow VAS score), *p* < 0.05 was considered that the difference was statistically significant. This study was a randomized controlled trial with a parallel design, PASS (Version 15.0, NCSS, Kaysville, UT, USA) was used to evaluate the sample size (*α* = 0.05, two‐sided). An estimated total of 65 patients would be needed to provide 80% power. Assumed loss of follow‐up rate was 10%, 83 patients was enough of a sample size for this study.

## Results

### Functional Results

All 83 patients were followed up, and the average hospital stay was 7.3 days. There was no significant difference in the preoperative general data between the two groups (*p* > 0.05, Table 1), which was comparable. There was no significant difference between the two groups in operation time, total length of incision, fracture healing time, Constant‐Murley shoulder function score, Mayo elbow function score and VAS pain score at the final follow‐up (*p* > 0.05, Table 2).

**TABLE 2:** Comparison of operation time, intraoperative bleeding, total length of incision, VAS pain score of shoulder and elbow, Constant score of shoulder and Mayo elbow in the last follow‐up between the study group and the control group.

Group	*N*	Surgery time (min)	Blood loss (mL)	Incision length (mm)	Healing time (Weeks)	VAS (Shoulder)	VAS (Elbow)	Constant score	Mayo score
A	40	130.90 ± 27.88	76.98 ± 16.46	18.2 ± 1.78	10.9 ± 2.1	2 (1,2)	2 (1,2)	82.77 ± 12.27	90.5 ± 8.5
B	43	119.39 ± 34.27	155.44 ± 54.98	16.6 ± 3.56	11.8 ± 3.8	1 (1,2)	2 (1,2)	85.15 ± 12.30	88.9 ± 10.1
Statistic and *p* value	‐	4.638	11.134	4.821	−10.897	1.481	1.182	1.318	20.443
	‐	0.208	<0.001	0.098	0.609	0.139	0.237	0.191	0.491

### Intraoperative Bleeding

The amount of intraoperative bleeding in the study group was significantly less than that in the control group (*p* < 0.01, Table 2).

### Complications

There were no radial nerve injury, musculocutaneous nerve injury, incision infection and fracture nonunion in the observation group. In the control group, there were four cases of iatrogenic radial nerve injury (all cases recovered after conservative treatment, three cases of incision infection (all cases recovered after conservative treatment) and three cases of fracture nonunion (all the patients received a second surgery and healed). The incidence of complications was 23.3% (10/43). There was significant difference between the two groups in the incidence of complications (*p* < 0.01, Table 3).

**TABLE 3:** Comparison of the incidence of complications and total complications between the study group and the control group.

Group	*N*	Radial N Injury	Musculocutaneous nerve injury	Infection	Unhealing	Total
A	40	0	0	0	0	0
B	43	4	0	3	3	10
Statistic and *p* value						10.577
						<0.001

## Discussion

### Main Findings

We developed a new method of treating distal third humeral shaft fracture. We used a humeral intramedullary nail combined with an anterior minimally invasive plate in the treatment of distal humeral shaft fracture, which has less complication and shorter surgery time. However, there was no difference in post‐operative function between the two groups.

In this study, there was no significant difference in the total length of surgical incision and operation time between group A and group B (*p* > 0.05), but group A had obvious advantages in the amount of bleeding and postoperative infection. Although the total length of incision is similar, the segmented small incision has a smaller range of soft tissue damage, so the amount of bleeding is less.

### Infection

Infection is a common complication in open humeral surgery, whose rate was about 2%–4%.^14^ At the same time, the vitality and integrity of soft tissue play an important role in the prevention of infection. Too much exposure means higher infection rates. In this study, there were no infections in group A, while there were three cases of infection in group B, which means the minimal method can significantly lower the possibility of infection.

### Radial Nerve Injury

Some studies have shown that the rate of iatrogenic radial nerve injury in the treatment of lower humeral shaft fractures by posterior approach plates is higher than humeral nails and anterior minimally invasive plates.^15^ Also the radial nerve injury rate is higher in the revision of nonunion or secondary internal fixation.^11^, ^16^ Compared with the traditional treatment, a humeral intramedullary nail and an anterior minimally invasive plate did not need to expose the radial nerve during the operation, which reduced the risk of iatrogenic radial nerve injury. In this study, all patients in group A had no symptoms of nerve injury, while four patients in group B had iatrogenic radial nerve paralysis, meaning that the new method could protect of radial nerve injury well.

### Limitations and Strengths of this Study

There are some limitations of this study. First, the number of patients included was small, also there is no biomechanical comparative study of two surgical methods. What is more, we did not compare the nail with the combination method. In the future, we will continue to improve relevant research and improve deficiencies. This new treatment method has many advantages. First, the combination could increase the strength of fixation, and also significantly reduce the complication rate of surgery. Also, blood loss was less in this new method, which means less invasive surgery.

## Conclusion

In this study, we combined a humeral intramedullary nail with an anterior minimally invasive plate in treating lower humeral shaft fractures, which has the advantage of less soft tissue injury, less intraoperative bleeding and low risk of iatrogenic radial nerve injury. It is an effective method for distal third humeral shaft fractures but still needs more study.

## Author Contributions

All authors had full access to the data in the study and take responsibility for the integrity of the data and the accuracy of the data analysis. Conceptualization, Renbin Li and Jianhai Chen; methodology, Yichong Zhang and Dengbang Su; investigation, Gang Fu, Shuyujiong Ke, and Jianlong Chen; formal analysis, Dengke Zhu and Jingxiang Wu; resources, Hui Ge; writing—original draft, Yichong Zhang and Gang Fu; writing—review and editing, Fengfei Lin and Yan Zhang; visualization, Shuyujiong Ke; supervision, Renbin Li; funding acquisition, Jianhai Chen and Renbin Li.

## Funding Information

This study was funded Fujian Provincial Clinical Medical Research Center for First Aid and Rehabilitation in Orthopaedic Trauma (2020Y2014); Fuzhou Trauma Medical Center (2018080303); Peking University People's Hospital Scientific Research and Development Funds (RDG2021‐01, RDL2021‐08). The Science and Technology Planning Project of Fuzhou (2022‐S‐053).

## Conflict of Interest

None.

## Ethics Statement

Institutional Review Board approval was received from the ethics committee of Fuzhou second hospital affiliated to Xiamen University (2020133) and all consents were signed to participate and publish.

## Authorship Declaration

All authors listed meet the authorship criteria according to the latest guidelines of the International Committee of Medical Journal Editors, and that all authors are in agreement with the manuscript.
